# enhancedGraphics: a Cytoscape app for enhanced node graphics

**DOI:** 10.12688/f1000research.4460.1

**Published:** 2014-07-01

**Authors:** John H. Morris, Allan Kuchinsky, Thomas E. Ferrin, Alexander R. Pico

**Affiliations:** 1 Resource for Biocomputing, Visualization and Informatics, University of California, San Francisco, CA 94143, USA; 2 Agilent Technologies, Santa Clara, CA 95051, USA; 3 Gladstone Institutes, San Francisco, CA 94158, USA

## Abstract

enhancedGraphics ( http://apps.cytoscape.org/apps/enhancedGraphics) is a Cytoscape app that implements a series of enhanced charts and graphics that may be added to Cytoscape nodes. It enables users and other app developers to create pie, line, bar, and circle plots that are driven by columns in the Cytoscape Node Table. Charts are drawn using vector graphics to allow full-resolution scaling.

## Introduction

Cytoscape ^1, 2^ provides support for coloring and sizing nodes and node borders based on data values stored in the Node Table. This provides an extremely useful mapping between data values and a single visual property, but does not solve the need for more complex visualizations. Over the years, there have been attempts to support more complex mappings of multiple data values onto node visuals in Cytoscape. These include GOlorize ^3^, which maps GO terms to pie charts on nodes; GenePro ^4^, which visualizes groups of nodes as pie charts; VistaClara ^5^, which adds bar graphs to represent expression data; and more recently MultiColoredNodes ^6^. Each of these plugins and apps implemented their own graph and chart capabilities that are not accessible to other apps and in some cases not applicable outside of specific types of analyses. We felt that a better approach would be to implement an app that provided general support for graphs and charts to users as well as to the developers of other apps. The initial version of this approach was developed for Cytoscape 2.8 as nodeCharts, which was used by clusterMaker ^7^, for example, and numerous users (see Figure 3 in the Jäger, *et al.* paper ^8^). For Cytoscape 3, we reimplemented this approach as an app to take advantage of the new architecture and custom graphics API. The mechanism supports saving and restoring charts, as well as high-quality image file output suitable for publication. The intent is to provide a single, consistent, mechanism to draw charts and graphs on nodes as a general solution for diverse users and other app developers, mitigating the need to reinvent this capability in future apps.

## Implementation

As part of the visual property mechanism, enhancedGraphics utilizes the Cytoscape 3 custom graphics API (org.cytoscape.view.presentation.customgraphics). To use the gradients and charts provided by enhancedGraphics, an app or user would create two things: a column that contains the instructions for creating the chart, and a passthrough visual mapping that maps that column to one of the custom graphics visual properties. The format of the instruction column is type: arglist, where type is the type of gradient or chart, and arglist is a list of name=value pairs that specify the arguments to create the gradient or chart (see details and examples in the tables below). The drawing and display of the chart or graph is handled by enhancedGraphics methods that are called by the Cytoscape rendering engine.

Internally, each enhancedGraphics chart type implements a *CyCustomGraphicsFactory* that is registered with OSGi ^9^. Each *CyCustomGraphicsFactory* informs the visual mapping mechanism of the chart type (e.g. **lingrad**) and method to create the *CyCustomGraphics* object given a String, which is the instruction column value. The *CyCustomGraphics* object parses the String as appropriate. Each *CyCustomGraphics* object implements a getLayers method that generates the appropriate list of *CustomGraphicLayers*. The API defines three types of *CustomGraphicsLayers*: (1) the base interface, *CustomGraphicsLayers*, that provides a getPaint method to return a simple java.awt.Paint for the node; (2) *ImageCustomGraphicLayer*, that adds a getPaint method that returns a java.awt.TexturePaint suitable for painting an image on a node; and (3) *PaintedShape* which adds methods to return Shapes, Strokes, and Paints to draw arbitrary shapes. enhancedGraphics utilizes the base *CustomGraphicsLayer* for the two gradient types and *PaintedShape* for all of the charts.

## Results

**Figure 1.**
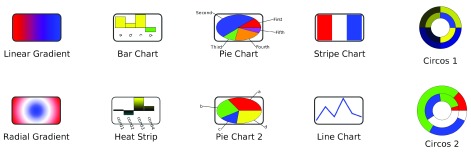
enhancedGraphics example Gradients and Charts.

Figure 1 shows examples of all of the gradients and charts that are provided by enhancedGraphics. Up to nine different graphs can be combined on a single node by mapping different columns to different Custom Graphics properties and then offsetting the charts using the corresponding Custom Graphics Position properties. enhancedGraphics currently provides two different types of graphics options: gradients and charts.

### Gradients

Gradients are simple paints on nodes. In order to provide the user with control over the exact paint to use, both linear and radial gradients allow the user to specify the gradient start and end (or center point and radius) and a stop list of color and opacity values. Table 1 provides the prefixes and arguments for the two gradient types.

**Table 1.** Gradient prefixes and arguments.

Type	Prefix	Argument	Description
Linear Gradient	**lingrad**	**start**=”x,y” **end**=”x,y” **stoplist**=”r,g,b,a,stop1|r,g,b,a,stop2|...”	x and y proportion (0-1) of where the gradient starts x and y proportion (0-1) of where the gradient ends red,green,blue, and opacity values (0–255) at each stop, which is interpreted as a proportion
Radial Gradient	**radgrad**	**center**=”x,y” **radius**=”r” **stoplist**=”r,g,b,a,stop1|r,g,b,a,stop2|...”	x and y proportion (0-1) of the gradient center proportional (0-1) radius red,green,blue, and opacity values (0–255) at each stop, which is interpreted as a proportion

### Charts

enhancedGraphics currently provides six chart types: bar, circos, heat strip, line, pie, and stripe. Each chart type has it’s own set of arguments as shown in Table 3. In addition, there are a number of common options that are used by many of the charts. Table 2 provides the syntax and explanation for each of these common arguments.

**Table 2.** Common arguments used by many charts.

Argument	Description
**attributelist**=”attr1,attr2,...attrn” **colorlist**=[ **contrasting**| **modulated**| **rainbow**| **random**| *updown colors*| *colors*] **labels**=”label1,label2,..., labeln” **labelcolor**= *color* **labelfont**= *font name* **labelsize**= *value* **labelstyle**=[ **italics**| **bold**| **bolditalic**| **plain**] **range**=” *min*, *max*” **scale**=” *scale*” **showlabels**=[ **true**| **false**] **values**=” *v1,v2,...vn*” **ybase**=[ **top**| **middle**| **bottom**| *value*]	List of columns to use to get the values for the chart. The **colorlist** argument provides a number of options, including a series of keywords for automatically generating colors. *updowncolors* is a specification of colors for positive, negative, and zero values: **up**: *color*, **down**: *color*[, **zero**: *color*] where the zero is optional. *colors* may be specified as a color name (red, green, blue) or an rgb or rgba color in hex notatation, e.g. #FF0000 for red. List of labels for the chart. If not provided and attributelist is provided, the names of the attributes are used as labels. Color of the labels Font to use for the labels Size of the label font label style The min and max range as floating point values. This is used to have consistent scaling across all nodes A floating point value used to scale the chart. If false, labels aren’t drawn A list of values to use for the chart. One of values or attributelist will often be required, but not both The specified the base of the chart. Usually used to set the location of the 0 value for line and bar graphs.

**Table 3.** Charts and arguments.

Chart Type	Prefix	Argument	Description
*Bar Chart* Simple bar chart. Multiple charts may be combined to get both up and down values. Accepts all of the common arguments.	**barchart**	**separation**= *value*	The separation between bars
*Circos chart* Circos plots (more properly donut or ring charts) use many of the standard values except **range**, **scale**, and **ybase**. Also note that **attributelist** should be a list of List attributes in you intend to have more than one ring.	**circoschart**	**arcstart**= *value* **arcwidth**= *value* **firstarc**= *value* **firstarcwidth**= *width* **labelcircles**= [ **true** | **false**] **sortslices**= [ **true** | **false**]	The start of each circle in degrees The thickness of each of the rings The start of the first arc as a proportion of the entire node The width of the first arc If true label each circle If true sort the slices from largest to smallest
*Heat strip chart* Heatstrip charts provide an up/ down bar graph with each bar colored as a gradient to reflect the values. *Colorlist* is interpreted differently for these charts	**heatstripchart**	**colorlist**= *gradient keyword*| *updown colors* **separation**= *value*	Current *gradient keywords* include: **yellowcyan**, **yellowblue**, **orangepurple**, **bluegreenyellow**, **purpleyellow**, **greenpurple**, **redyellow**, and if you absolutely must: **redgreen**. See Table 2 for a description of *updown colors*. The separation between bars
Line chart Simple line graph. Accepts the standard arguments	**linechart**	**linewidth**= *value*	The width of the lines on the plot
*Pie chart* Simple pie chart. Accepts all standard values except textbfrange, **scale**, and **ybase**. *Stripe chart* Very simple chart that breaks the node into n colors determined by the colorlist argument. No other arguments are used.	**piechart** **stripechart**	**arcstart**= *value* **sortslices**= [ **true** | **false**]	The start of each circle in degrees If true sort the slices from largest to smallest

### Examples

The example charts shown in Figure 1 and provided in the Cytoscape session file Supplementary File 1 are generated from data columns. The instructions in the chart columns assume that the following columns exist: a, b, c, and d are integer columns in the default node table; Values is a list of Doubles also in the default node table, and Circle1 and Circle2 are also lists of Doubles. At this point, gradients are not dependent on any internal data. See Supplementary File 1 to see the instructions that generated Figure 1.

**Figure 2.**
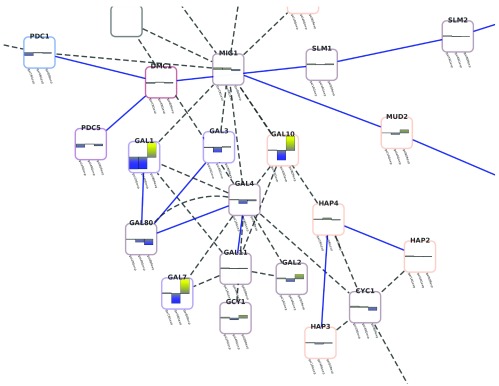
Example of using enhancedGraphics to show expression data in the context of a protein-protein interaction network.

A more relevant biological example is shown in Figure 2. This image shows a portion of the galFiltered.cys network delivered as part of the sampleData with every Cytoscape download. The bar charts show the values of the expression data included as columns gal1RGexp, gal4RGexp, and gal80Rexp. A string column was created and all rows were filled with the enhancedGraphic arguments:



                        heatstripchart:
    attributelist="gal1RGexp,gal4RGexp,gal80Rexp"
    colorlist="yellowblue" range="-3.0,3.0"
                    


## Conclusions

enhancedGraphics fills an important need for Cytoscape visualizations: the ability to display more complicated data relationships as graphical representations onto nodes. enhancedGraphics has been integrated into clusterMaker to show heatstrips on nodes corresponding to clusters, into upcoming apps such as cddApp, which connects to the NCBI CDD Database and uses enhancedGraphics to show pie charts of the domain coverage. We have also used enhancedGraphics to show sequence coverage histograms on nodes that represent sequence contigs. In the future, we want to improve the font handling and add support for small heatmaps painted on nodes. We also plan to add a graphical interface to help users construct these visualizations without having to write out instruction arguments. The concise syntax, however, will still be valuable to advanced users and other app developers seeking to create enhanced graphics. With enhancedGraphics, Cytoscape users and app developers can visualize multiple columns of data as graphs and charts on their network nodes.

## Software availability

Software available from: http://apps.cytoscape.org/apps/enhancedGraphics

Latest source code: https://github.com/RBVI/enhancedGraphics

Source code as at the time of publication: https://github.com/F1000Research/enhancedGraphics/releases/tag/v1

Archived source code as at the time of publication: http://www.dx.doi.org/10.5281/zenodo.10421 ^11^

Software license:

Lesser GNU Public License 3.0: https://www.gnu.org/licenses/lgpl.htmlM

## Supplementary material

Example of the Instructions used to create Figure 1.
