# Supplementary material for: enhancedGraphics: a Cytoscape app for enhanced node graphics
Source: F1000Res. 2014 Jul 1;3:147. [Version 1] doi: 10.12688/f1000research.4460.1 (PMC4176421; doi:10.12688/f1000research.4460.1)
Supplement: Peer review history

# Peer review: Referee response for version 1

**DOI:** 10.5256/f1000research.4771.r6001

**Published:** 2014-09-04

**Authors:** Piet Molenaar

**Affiliations:** 1 Academic Medical Center, Amsterdam, Netherlands

The app described in the article addresses a specific issue with the Cytoscape network visualization tool: drawing of multidimensional data on nodes is currently not possible by default. As such this is a very valuable addition to the Cytoscape app ecosystem. The article is well written but I do have some minor issues that can be considered for further improvement:

**Minor issue**

The implementation details are a too technical when an intended audience includes biologists (and might even scare them away). I would suggest to transfer these OSGI specific details to a supplement or add a protocol section before this section with a sample protocol (including installation details).

**Minor issue**

The current sample session file included in the supplemental data only contains example nodes. A session file or additional network showing actual data mapped on the gal dataset (as in the figure) would further clarify the actual usage of the app.

**Minor issue**

In the conclusions section references to cddApp and NCBI CDD Database are lacking.

I have read this submission. I believe that I have an appropriate level of expertise to confirm that it is of an acceptable scientific standard.

# Peer review: Referee response for version 1

**DOI:** 10.5256/f1000research.4771.r5308

**Published:** 2014-07-17

**Authors:** Lars Juhl Jensen

**Affiliations:** 1 NNF Center for Protein Research, University of Copenhagen, Copenhagen, Denmark

**General comments:**

The authors describe a Cytoscape App, which provides a generic interface for displaying additional data onto the nodes of a graph. I believe that this App will be highly useful to researchers working with transcriptomics and proteomics data, as these often have a need to display, for example, expression time-courses onto protein interaction networks. This is especially true if the App is used as the foundation for other more specialized Apps that make it easier for users to import and visualize specific data types in an appropriate manner.

**Specific comments:**

The *Integration* section is difficult to fully follow for people who are not Cytoscape developers, who cannot be assumed to know what, for example, a *CyCustomGraphicsFactory* is. It would in my opinion be good to revise this section to make it understandable to a broader audience.

In Figure 1, I find it strange that the pie charts are not circular and that the circos charts, unlike all the other chart types, are not embedded within a frame with rounded corners. Also, the figure would benefit from a figure caption explaining the difference between Pie Chart 1 and Pie Chart 2, as well as between Circos 1 and Circos 2. The differences between these chart subtypes are not clear to me.

I have read this submission. I believe that I have an appropriate level of expertise to confirm that it is of an acceptable scientific standard.
